# Supplementary material for: Structure of the error-prone DNA ligase of African swine fever virus identifies critical active site residues
Source: Nat Commun. 2019 Jan 23;10:387. doi: 10.1038/s41467-019-08296-w (PMC6344480; doi:10.1038/s41467-019-08296-w)
Supplement: Supplementary file 1 — Supplementary Information [file 41467_2019_8296_MOESM1_ESM.pdf]

Supplementary Information

For

Structure of the error-prone DNA ligase of African swine fever virus identifies critical active site residues

Yiqing Chen[1], Hehua Liu[1,2], Chun Yang[1], Yanqing Gao[1], Xiang Yu[1,2], Xi Chen[1], Ruixue Cui[2], Lina Zheng[2], Suhua Li[1], Xuhang Li[2], Jinbiao Ma[2], Zhen Huang[3,4,*], Jixi Li[1,5*], Jianhua Gan[1,*]

[1]State Key Laboratory of Genetic Engineering, Collaborative Innovation Center of Genetics and Development, Department of Physiology and Biophysics, School of Life Sciences, Fudan University, Shanghai, 200433, China

[2]State Key Laboratory of Genetic Engineering, Collaborative Innovation Center of Genetics and Development, Department of Biochemistry, School of Life Sciences, Fudan University, Shanghai, 200433, China

[3]College of Life Sciences, Sichuan University, Chengdu, 610041, China

[4]Department of Chemistry, Georgia State University, Atlanta, GA 30303, USA

[5]Department of Neurology, Huashan Hospital, Fudan University, Shanghai, 200040, China

***Correspondence to**: ganjhh@fudan.edu.cn or lijixi@fudan.edu.cn or huang@gsu.edu

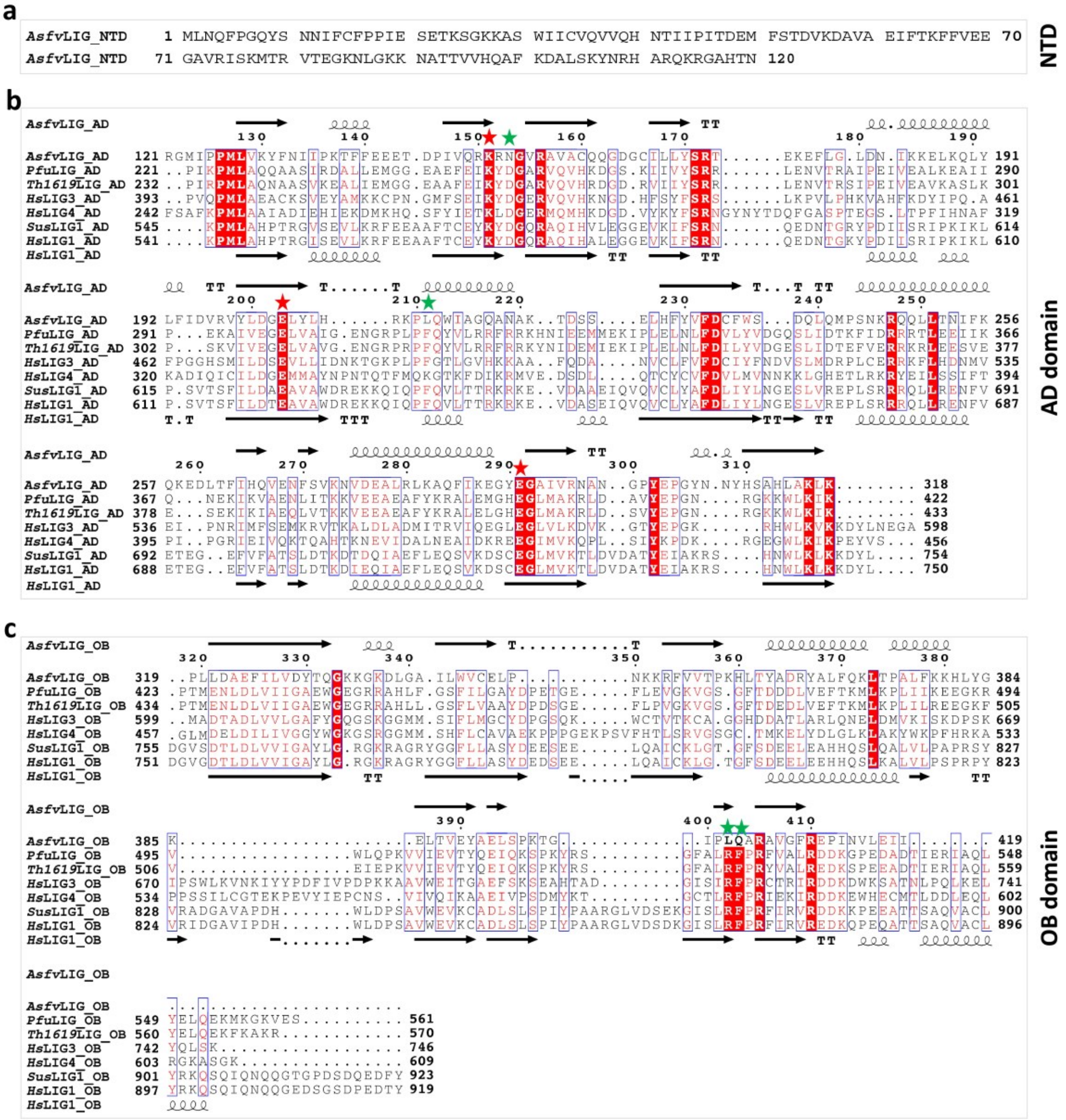

Supplementary Fig. 1 Sequence of *Asfv*LIG and alignment with homologous proteins. **a** Sequence of *Asfv*LIG NTD domain. **b** Sequence alignment of the AD domains. **c** Sequence alignment of the OB domains. *Asfv, African swine fever virus; Pfu, Pyrococcus furiosus; Th1519, Thermococcus sp. 1519; Sus, Sus scrofa; Hs, Homo sapiens*. The secondary structures of *Asfv*LIG and *Hs*LIG1 are shown on the top and at bottom, respectively. The catalytic residues and residues in the proximity of the nick of the DNA substrate are indicated by red and green asterisks, respectively.

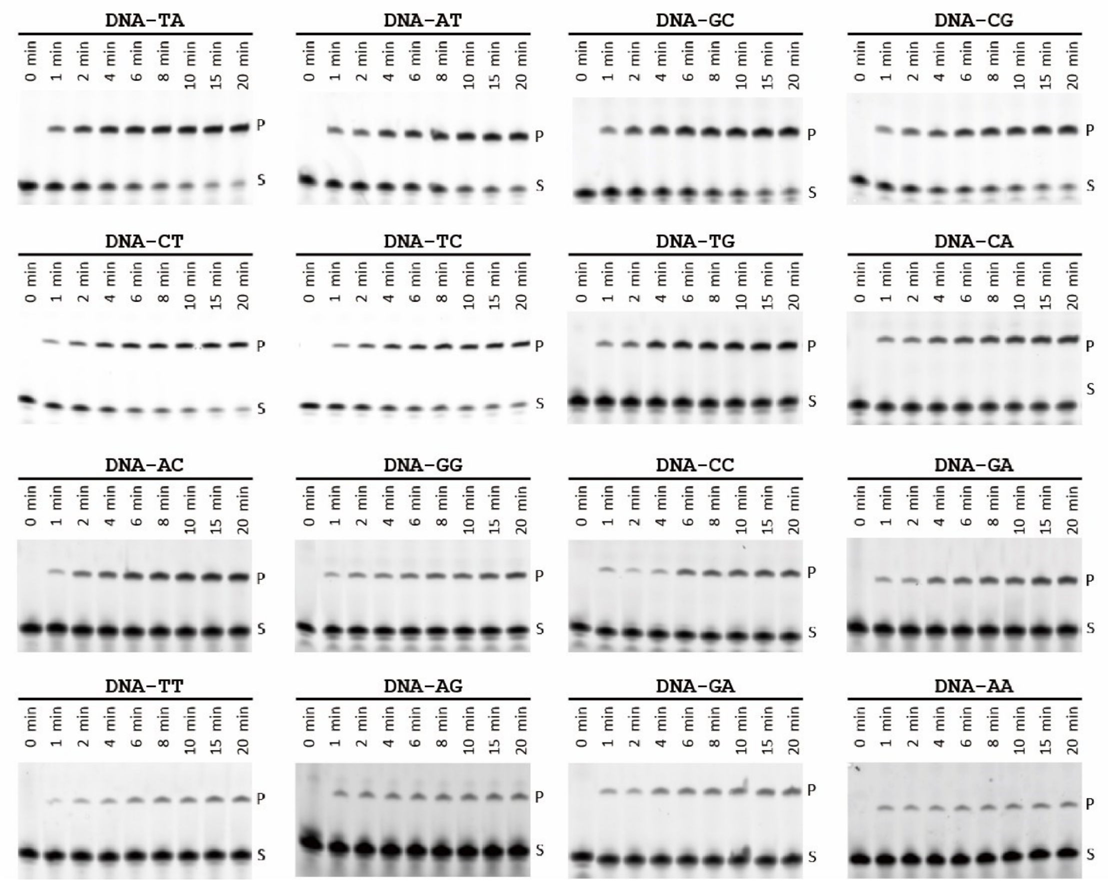

Supplementary Fig. 2 *In vitro* DNA ligation catalyzed by WT *Asfv*LIG. The substrates are named as DNA-XY, where X and Y denote the nucleotides at the template strand and at the 3'-end of the upstream of the nick, respectively. The substrate and product bands are labelled as S and P, respectively.

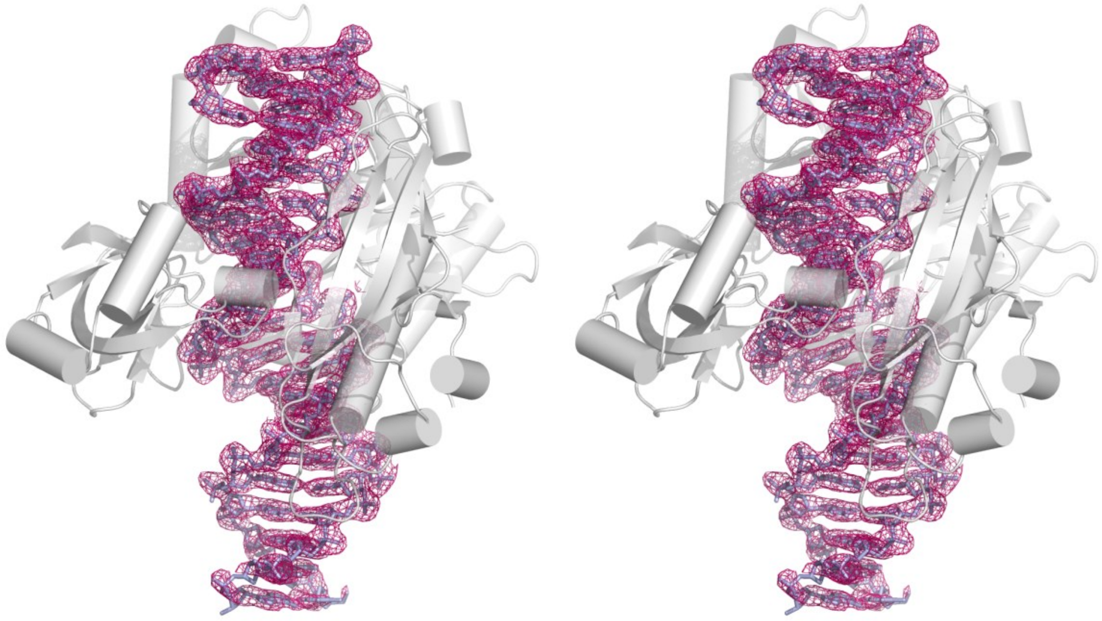

Supplementary Fig. 3 Stereo-view showing the overall structure of the catalytic form *Asfv*LIG:CT1 complex. *Asfv*LIG is shown as cartoon in white. DNAs are shown as sticks in light blue. The $2F_o$-F_c electron density maps of the DNAs are colored in magenta and contoured at 1.2 sigma level.

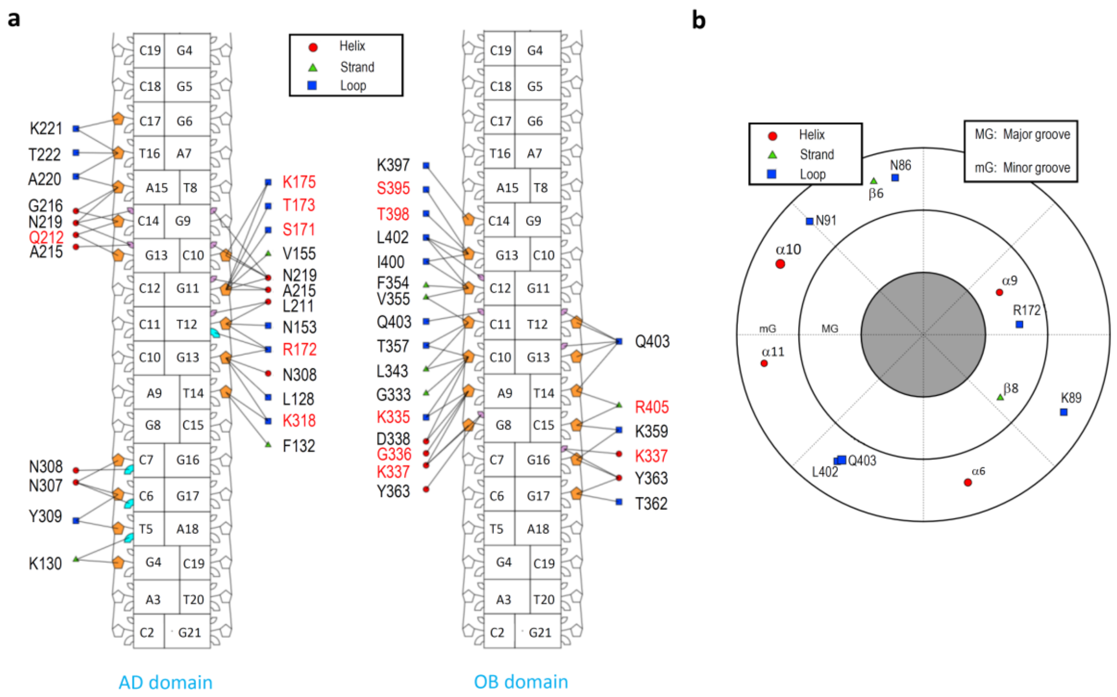

Supplementary Fig. 4 DNA-protein interactions observed in the catalytic form *Asfv*LIG:DNA complex. **a** Nucleotide-residue contact map showing individual nucleotide-residues interactions for the preferred binding site. Small and large markers on each nucleotide represent the major and minor groove contacts, respectively. Filled-in cyan and pink markers highlight which nucleotides are contacted by at least one residue in the major and minor groove, respectively. Residues that form conserved interaction with DNA in other ligase structures are labelled in red. **b** *Polar contact map* for the catalytic *Asfv*LIG:CT1 complex. Helix α9 and strand β8 bind in DNA major groove (MG, inner circle), whereas strand β6 and helices α6, α10, and α11 all bind in the minor groove (mG, outer circle). Arg172 contacts the major groove, but other loop residues, including Asn86, Lys89, Asn91, Leu402, and Gln403 all contact the DNA from the minor groove side.

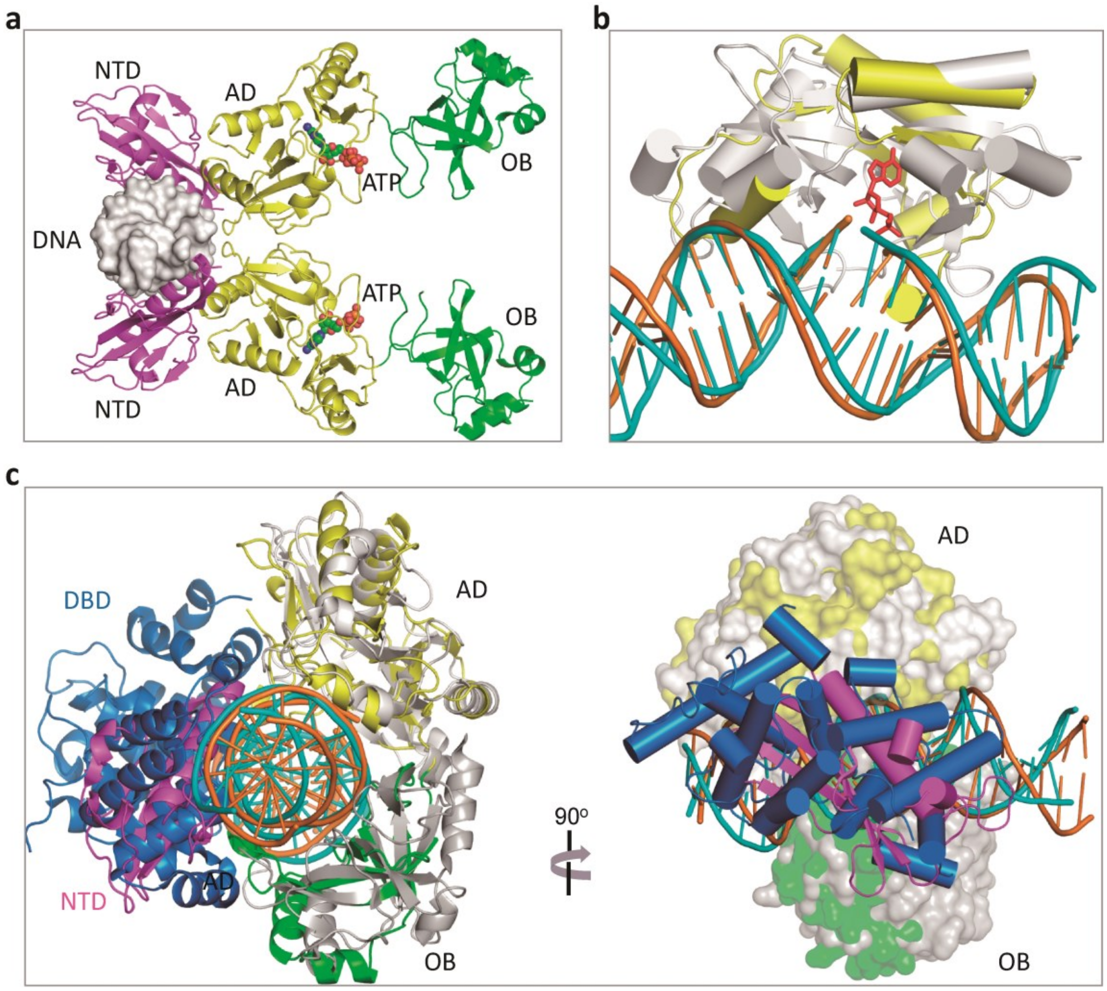

Supplementary Fig. 5 Comparison of *Asfv*LIG and *Hs*LIG1 structures. **a** The non-catalytic form *Asfv*LIG structure showing regular B-form DNA bound by two NTD domains. DNA is shown as surface in white. *Asfv*LIG is shown as cartoon with the NTD, AD, and OB domains colored in magenta, yellow, and green, respectively. ATP molecules are shown as spheres. **b** Structural superposition showing the similar nick site DNA bending. For clarity, only the AD domains are shown for the *Asfv*LIG (yellow) and *Hs*LIG1 (white) structures. DNA is shown as an orange cartoon for the *Asfv*LIG structure and a cyan cartoon for the *Hs*LIG1 structure. AMP that pyrophosphate linked to the 5'-P of downstream DNA is shown as red sticks. **c** Structural superposition showing the similar orientations but different folds of the NTD and DBD domains. DNA and the NTD, AD, and OB domains of *Asfv*LIG are colored in orange, magenta, yellow, and green, respectively. For the *Hs*LIG1 structure, DNA, the DBD, AD, and OB domains are colored in cyan, blue, white, and grey, respectively.

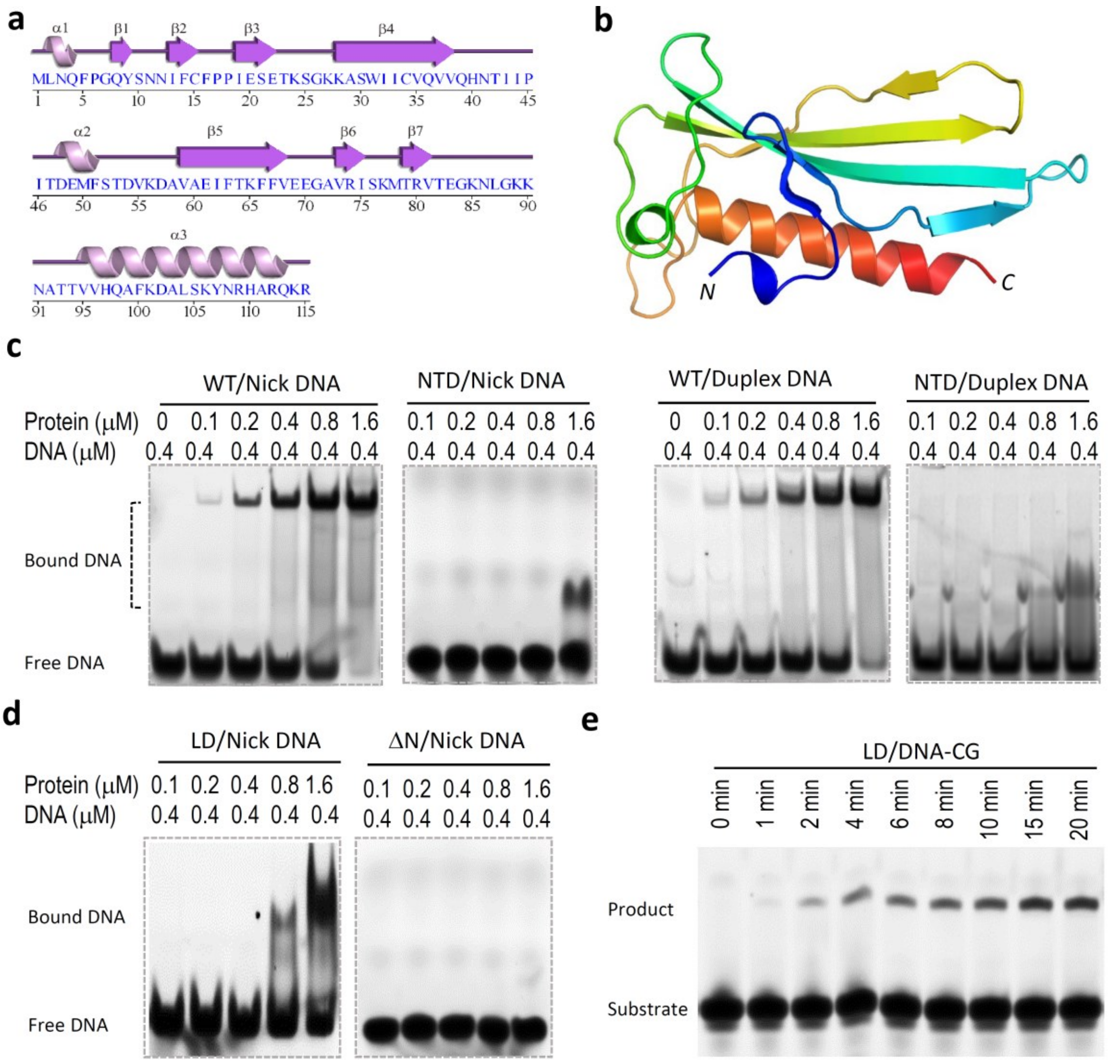

Supplementary Fig. 6 Structure of *Asfv*LIG NTD domain. **a** Sequence and secondary structure of *Asfv*LIG NTD. **b** Overall structure of *Asfv*LIG NTD. **c** *In vitro* nick and duplex DNA-CG binding by WT *Asfv*LIG, and AsfvLIG NTD. **d** *in vitro* nick DNA-CG binding by *Asfv*LIG with NTD residues 85-92 replaced with two Gly residues (for *Asfv*LIG LD) or *Asfv*LIG with NTD deleted (for *Asfv*LIG ΔN). **e** *In vitro* nick DNA-CG ligation by *Asfv*LIG LD.

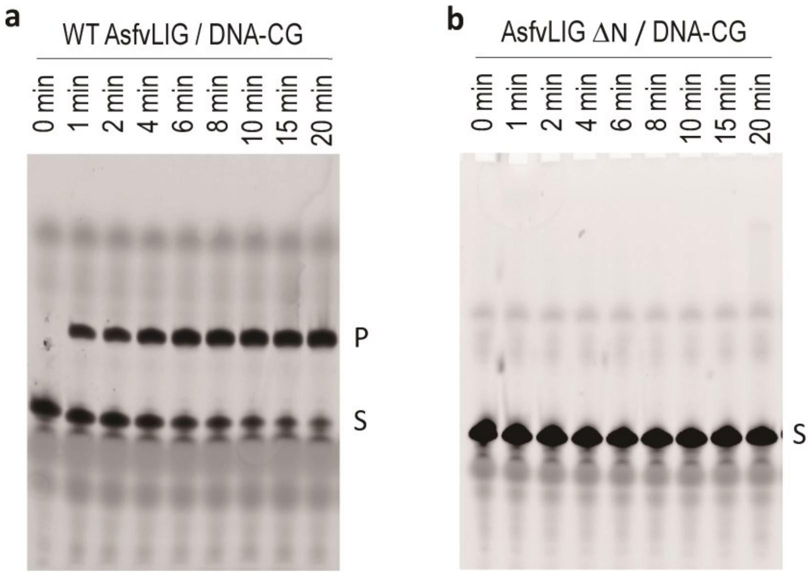

Supplementary Fig. 7 The uncropped gel images showing *in vitro* nick DNA-CG ligation by **a** WT *Asfv*LIG and **b** *Asfv*LIG ΔN, respectively. The substrate and product bands are labelled as S and P, respectively.

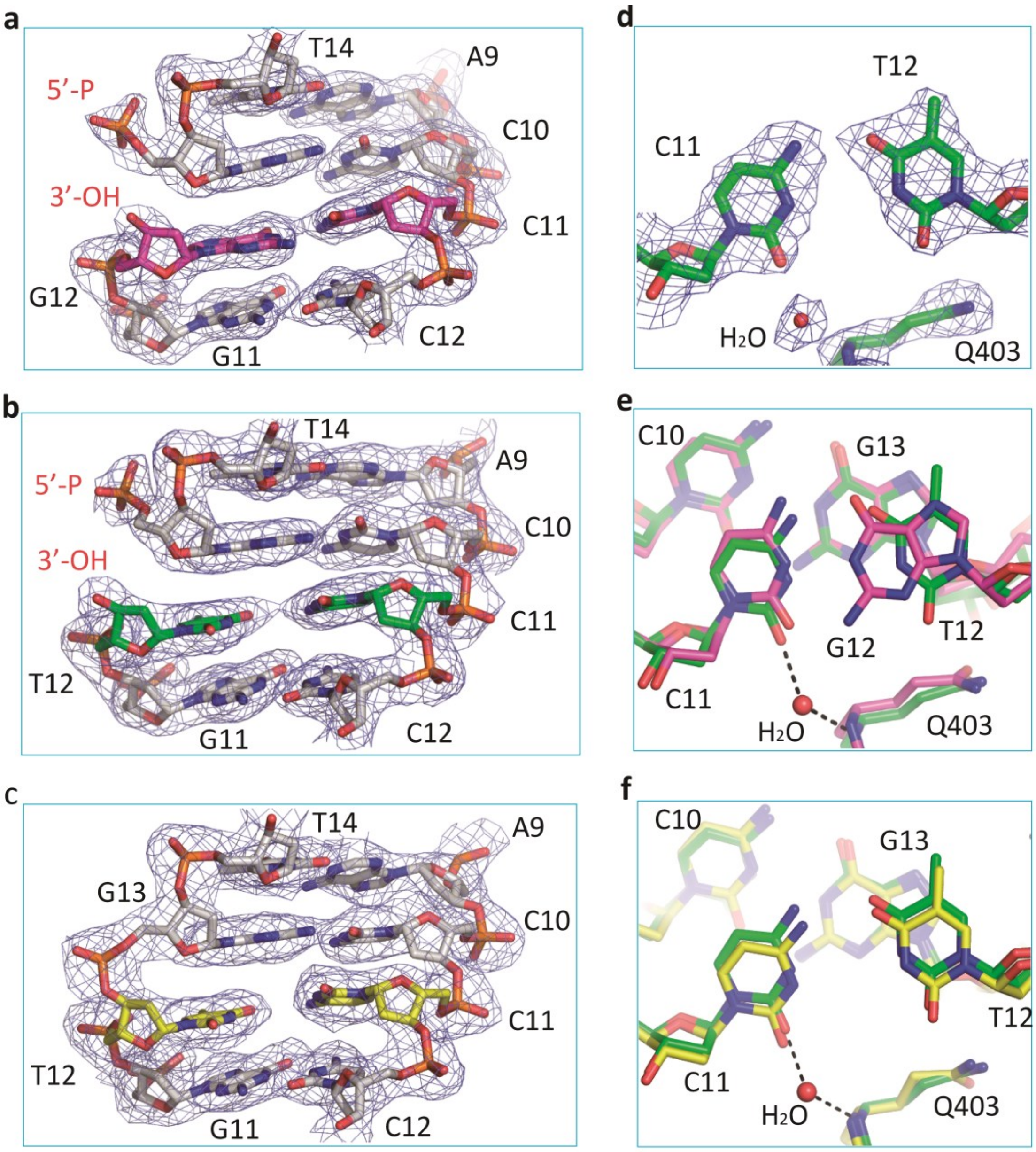

Supplementary Fig. 8 Conformational comparison of the nick site base pairs. Conformations of the base pairs located at the nick sites and flanking regions of **a** *Asfv*LIG:CG, **b** *Asfv*LIG:CT1, and **c** *Asfv*LIG:CT2 structures, respectively. **d** Local conformation of the C:T pair observed in the *Asfv*LIG:CT1 structure. **e** Superposition of the C:G and C:T pairs observed in the *Asfv*LIG:CG and *Asfv*LIG:CT1 structures, respectively. **f** Comparison of the C:T pairs observed in the *Asfv*LIG:CT1 and *Asfv*LIG:CT2 structures. In panels **a-c**, the $2F_o$-F_c electron density maps are all contoured at the 1.5 sigma level. C-atoms of the nick site C:G, C:T, and C:T base pairs are colored in magenta, green, and yellow for the *Asfv*LIG:CG, *Asfv*LIG:CT1 and *Asfv*LIG:CT2 structures, respectively.

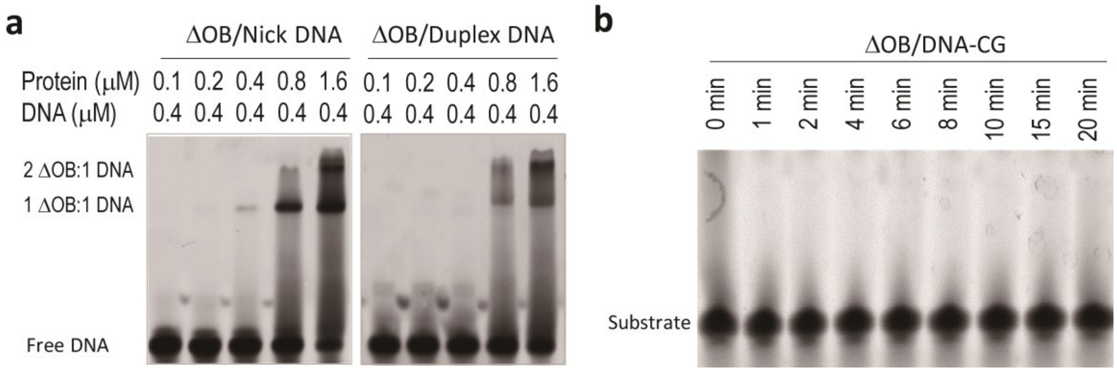

Supplementary Fig. 9 *In vitro* **a** DNA binding and **b** DNA ligation by *Asfv*LIG ΔOB protein. In the *Asfv*LIG ΔOB protein, the OB domain was deleted.

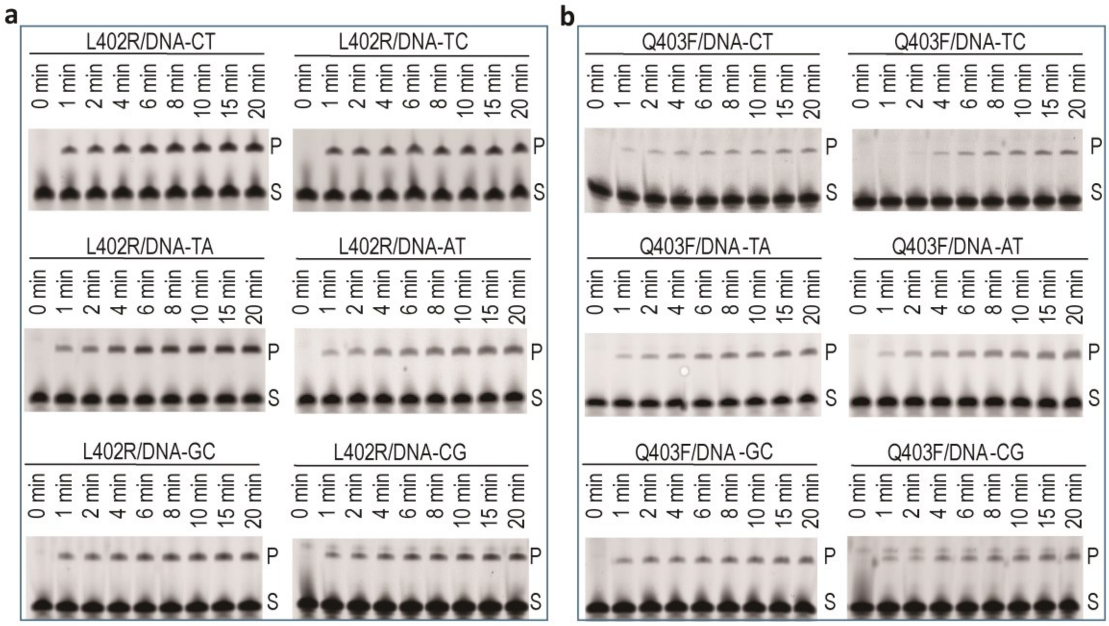

Supplementary Fig. 10 *In vitro* DNA ligation catalyzed by **a** L402R and **b** Q403F mutants of *Asfv*LIG. The substrate and product bands are labelled as S and P, respectively.

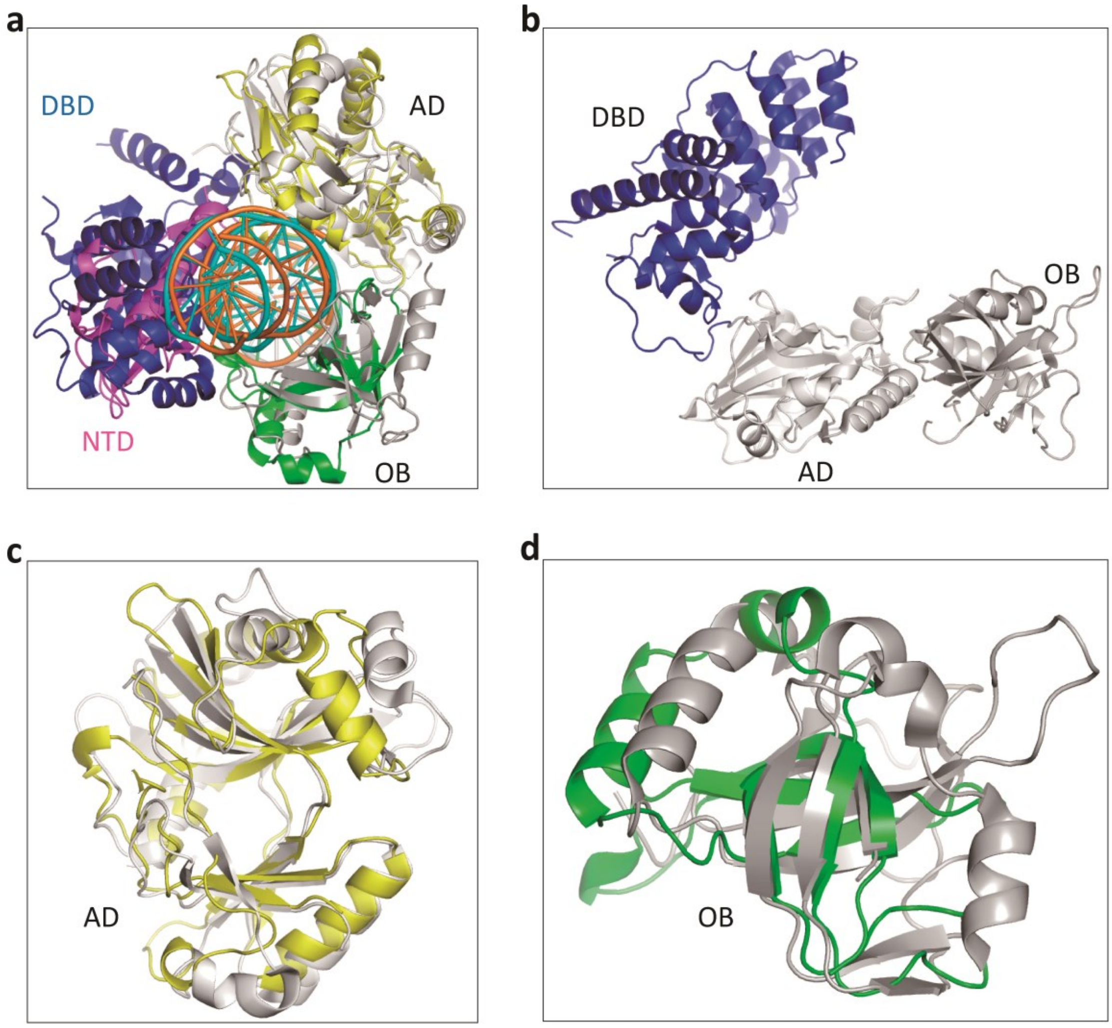

Supplementary Fig. 11 Comparison of *Asfv*LIG with the homologous proteins. **a** Structural superposition between *Asfv*LIG:CT1 complex and *Hs*LIG3:DNA complex (PDB_ID: 3L2P). For the *Asfv*LIG:CT1 complex, DNA and the NTD, AD, and OB domains are colored in orange, magenta, yellow, and green, respectively. For the *Hs*LIG3:DNA complex, DNA, the DBD, AD, and OB domains are colored in cyan, blue, white, and grey, respectively. **b** The open form *Hs*LIG4 structure (PDB_ID: 3W5O). **c** Comparison of the AD domains of *Asfv*LIG and *Hs*LIG4, which are colored in yellow and white, respectively. **d** Comparison of the OB domains of *Asfv*LIG and *Hs*LIG4, which are colored in green and grey, respectively.

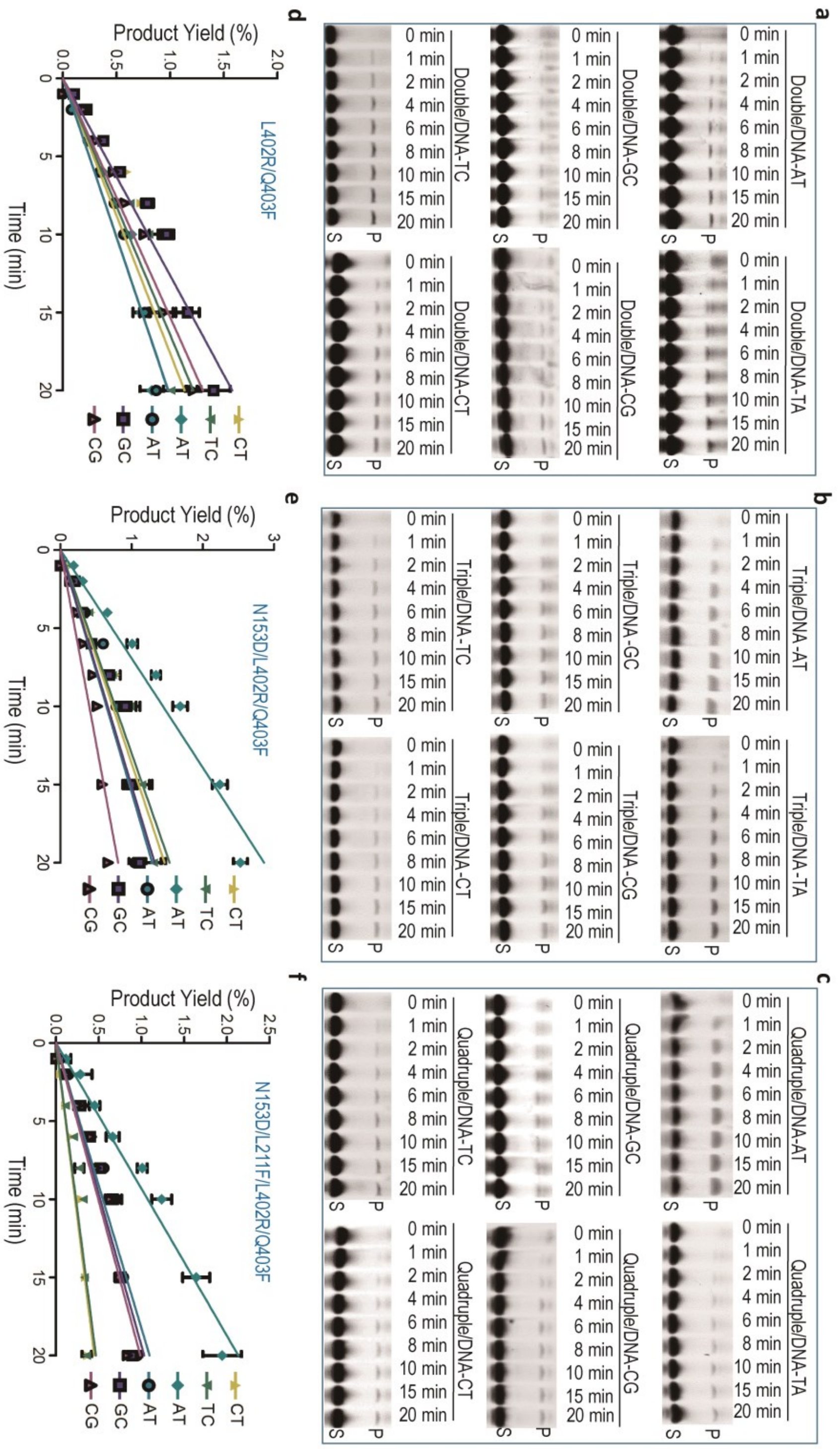

Supplementary Fig. 12 Gel analysis and quantification of *in vitro* DNA ligation catalyzed by the double mutant (L402R/Q403F, **a** and **d**), the triple mutant (N153D/L402R/Q403F, **b** and **e**), and the quadruple mutant (N153D/L211F/L402R/Q403F, **c** and **f**) of *Asfv*LIG. The substrate and product bands are labelled as S and P, respectively. In panels **d**-**f**, the data represent the mean of three independent experiments, with standard deviation (±SD) values indicated by error bars.

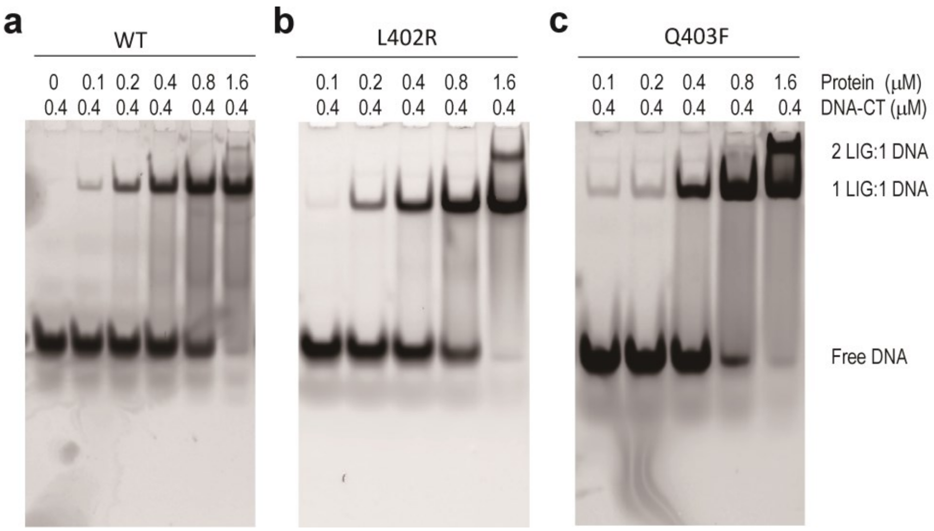

Supplementary Fig. 13 The uncropped gel images showing *in vitro* nick DNA-CG binding by **a** WT *Asfv*LIG, **b** L402R mutant, and **c** Q403F mutant, respectively.

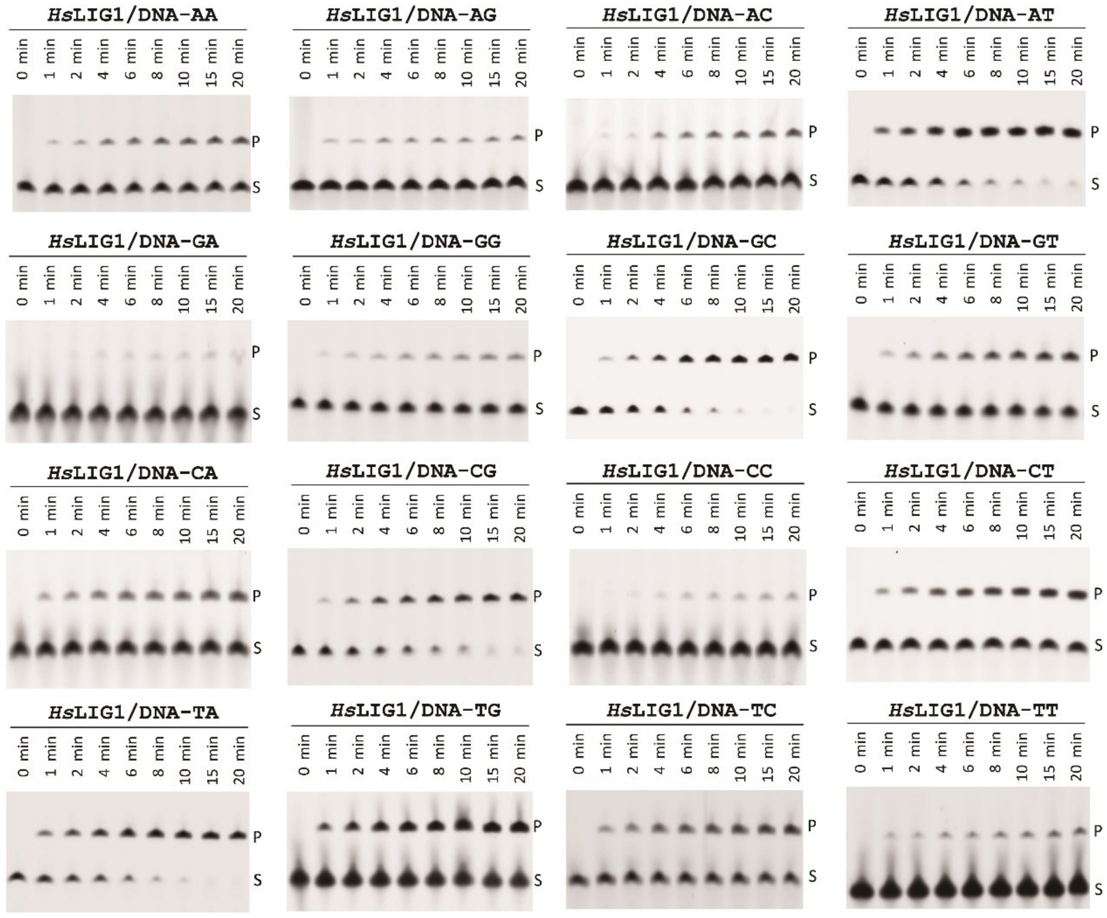

Supplementary Fig. 14 *In vitro* DNA ligation catalyzed by WT *Hs*LIG1. The substrate and product bands are labelled as S and P, respectively.

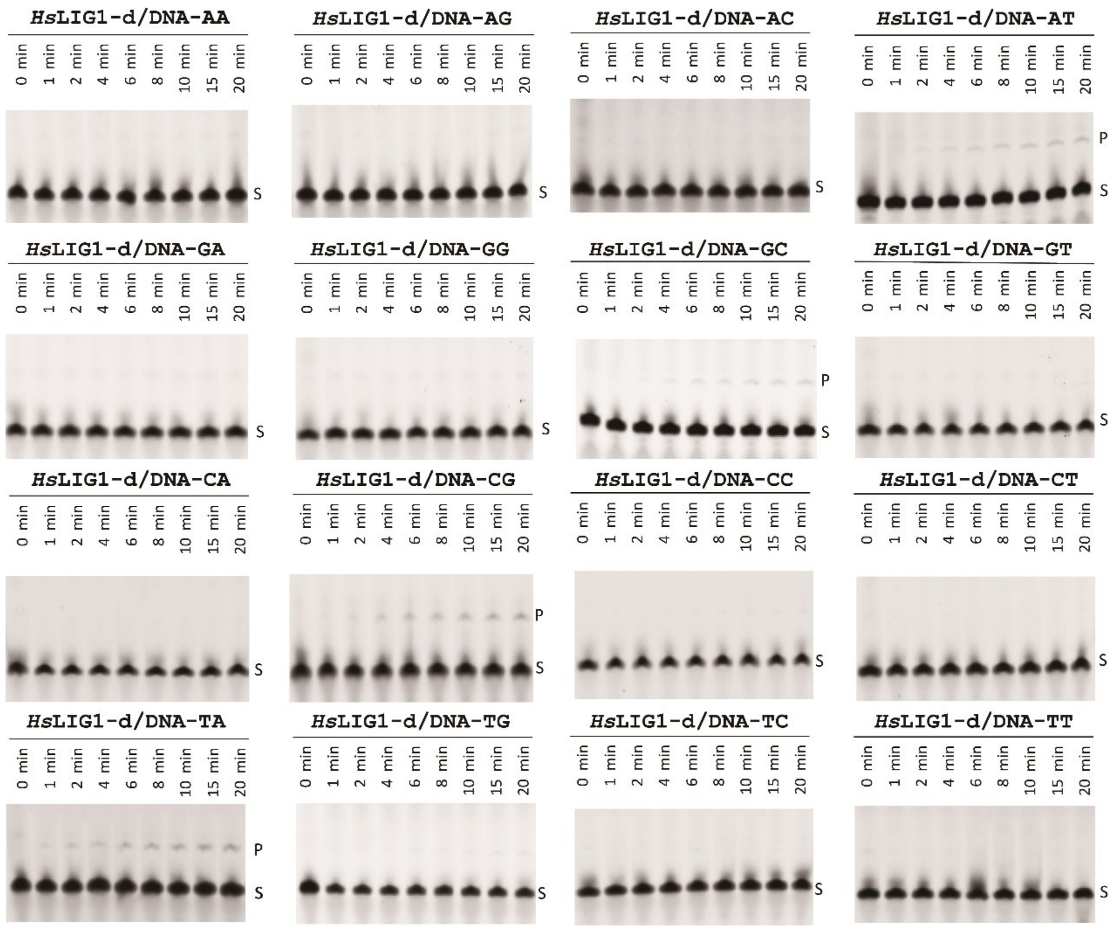

Supplementary Fig. 15 *In vitro* DNA ligation catalyzed by the *Hs*LIG1 R871L/F872Q double mutant (*Hs*LIG1-d). The substrate and product bands are labelled as S and P, respectively.

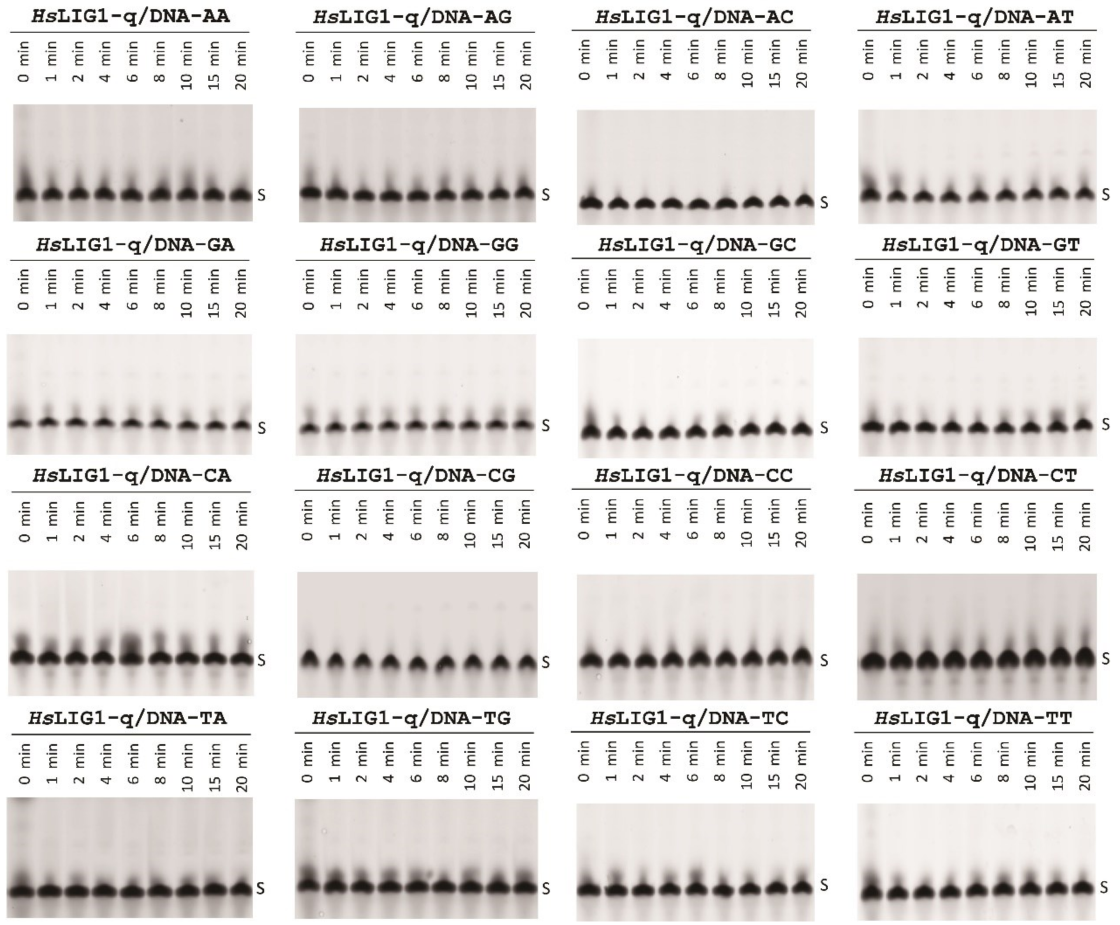

Supplementary Fig. 16 *In vitro* DNA ligation catalyzed by the *Hs*LIG1 D570N/F635L/R871L/F872Q quadruple mutant (*Hs*LIG1-q). The substrate bands are labelled as S.

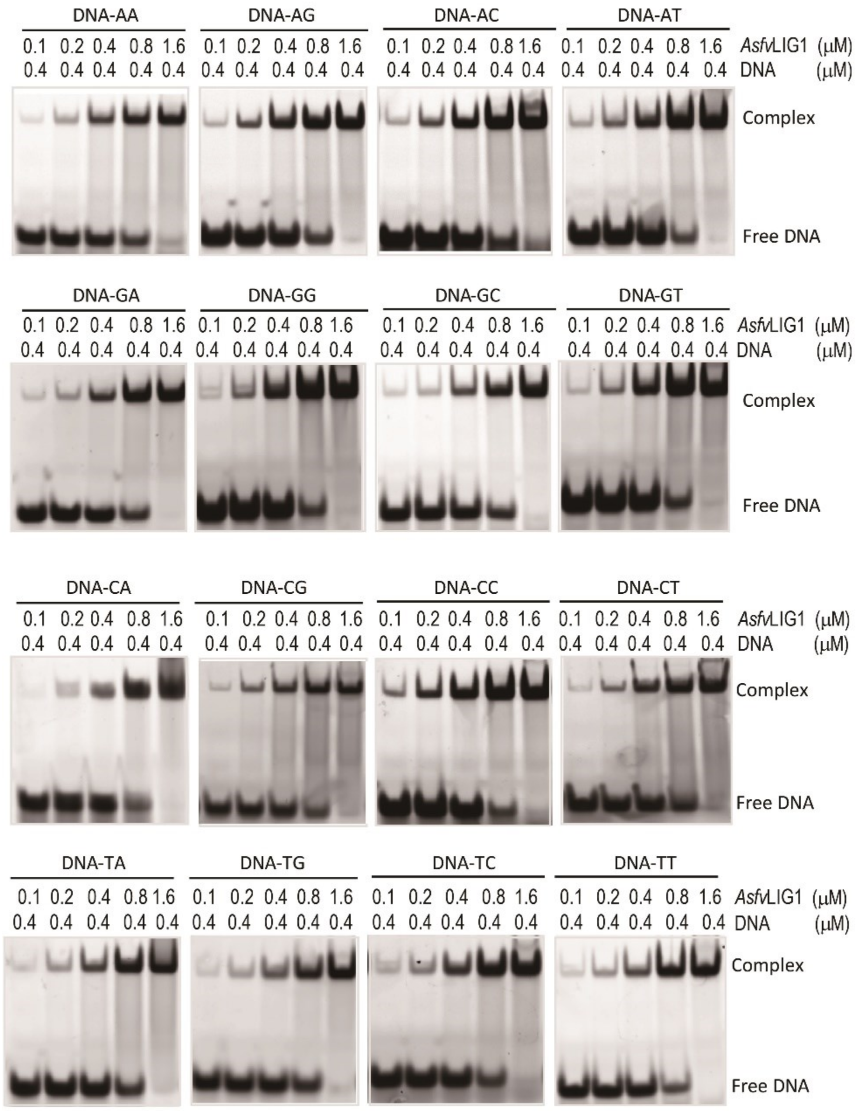

Supplementary Fig. 17 *In vitro* DNA binding by *Asfv*LIG1.

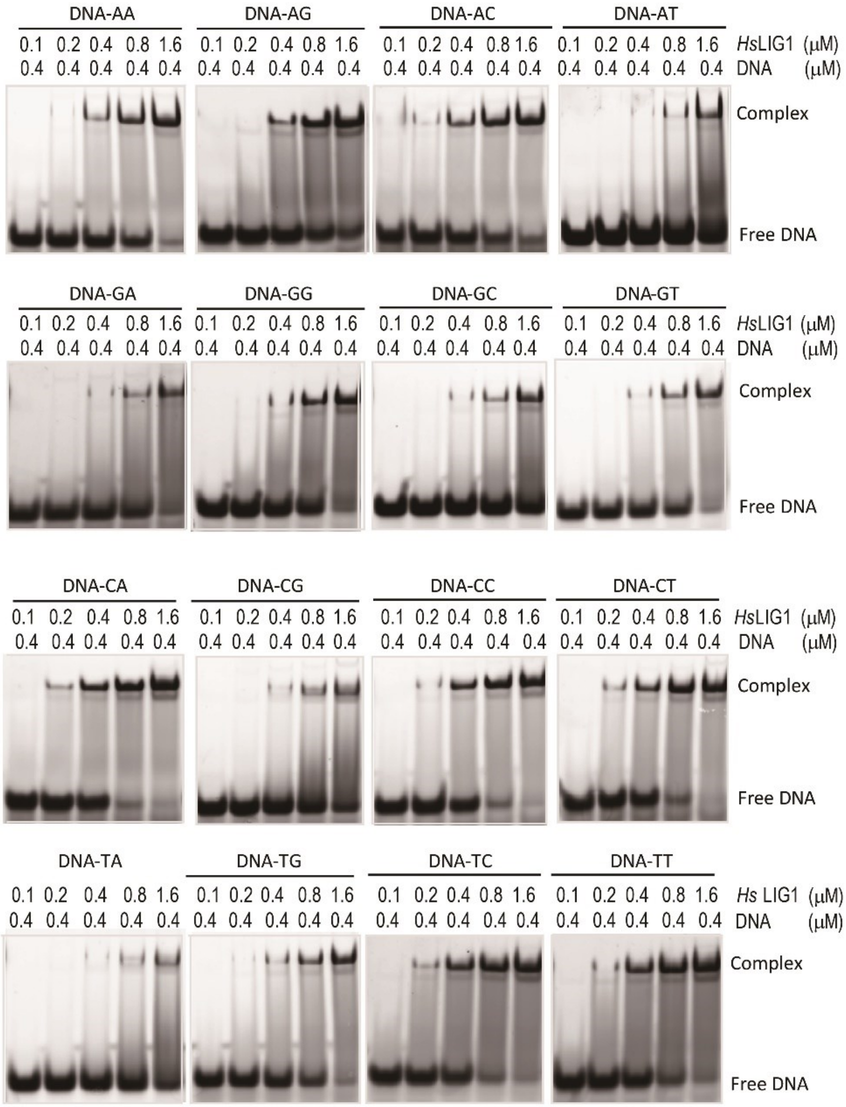

Supplementary Fig. 18 *In vitro* DNA binding by *Hs*LIG1.

Supplementary Table 1. Sequences of DNAs utilized in the *in vitro* binding and catalysis

<table>
<tr><th>Name</th><th>Sequence and structure [a]</th><th>Name</th><th>Sequence and structure [a]</th></tr>
<tr><td>DNA-AT</td><td>5′-CAGTCCGACACGCATCCCG-3′
|||||||||||||||||||
3′-GTCAGGCTGTGCGTAGGGC-FAM-5′
|
P</td><td>DNA-TA</td><td>5′-CAGTCCGACTCGCATCCCG-3′
|||||||||||||||||||
3′-GTCAGGCTGAGCGTAGGGC-FAM-5′
|
P</td></tr>
<tr><td>DNA-GC</td><td>5′-CAGTCCGACGCGCATCCCG-3′
|||||||||||||||||||
3′-GTCAGGCTGCGCGTAGGGC-FAM-5′
|
P</td><td>DNA-CG</td><td>5′-CAGTCCGACCCGCATCCCG-3′
|||||||||||||||||||
3′-GTCAGGCTGGGCGTAGGGC-FAM-5′
|
P</td></tr>
<tr><td>DNA-AA</td><td>5′-CAGTCCGACACGCATCCCG-3′
|||||||||¦|||||||||
3′-GTCAGGCTGAGCGTAGGGC-FAM-5′
|
P</td><td>DNA-GA</td><td>5′-CAGTCCGACGCGCATCCCG-3′
|||||||||¦|||||||||
3′-GTCAGGCTGAGCGTAGGGC-FAM-5′
|
P</td></tr>
<tr><td>DNA-AG</td><td>5′-CAGTCCGACACGCATCCCG-3′
|||||||||¦|||||||||
3′-GTCAGGCTGGGCGTAGGGC-FAM-5′
|
P</td><td>DNA-GG</td><td>5′-CAGTCCGACGCGCATCCCG-3′
|||||||||¦|||||||||
3′-GTCAGGCTGGGCGTAGGGC-FAM-5′
|
P</td></tr>
<tr><td>DNA-AC</td><td>5′-CAGTCCGACACGCATCCCG-3′
|||||||||¦|||||||||
3′-GTCAGGCTGCGCGTAGGGC-FAM-5′
|
P</td><td>DNA-GT</td><td>5′-CAGTCCGACGCGCATCCCG-3′
|||||||||¦|||||||||
3′-GTCAGGCTGTGCGTAGGGC-FAM-5
|
P</td></tr>
<tr><td>DNA-TG</td><td>5′-CAGTCCGACTCGCATCCCG-3′
|||||||||¦|||||||||
3′-GTCAGGCTGGGCGTAGGGC-FAM-5′
|
P</td><td>DNA-CA</td><td>5′-CAGTCCGACCCGCATCCCG-3′
|||||||||¦|||||||||
3′-GTCAGGCTGAGCGTAGGGC-FAM-5′
|
P</td></tr>
<tr><td>DNA-TT</td><td>5′-CAGTCCGACTCGCATCCCG-3′
|||||||||¦|||||||||
3′-GTCAGGCTGTGCGTAGGGC-FAM-5′
|
P</td><td>DNA-CC</td><td>5′-CAGTCCGACCCGCATCCCG-3′
|||||||||¦|||||||||
3′-GTCAGGCTGCGCGTAGGGC-FAM-5′
|
P</td></tr>
<tr><td>DNA-TC</td><td>5′-CAGTCCGACTCGCATCCCG-3′
|||||||||¦|||||||||
3′-GTCAGGCTGCGCGTAGGGC-FAM-5′
|
P</td><td>DNA-CT</td><td>5′-CAGTCCGACCCGCATCCCG-3′
|||||||||¦|||||||||
3′-GTCAGGCTGTGCGTAGGGC-FAM-5′
|
P</td></tr>
</table>

[a]: The continuous template strand is listed at the top of each DNA structure. The broken strand is listed at the bottom; the upstream is FAM-labelled and the downstream contains one phosphate group at their 5'-ends, respectively. The nick site base pairs are highlighted in red in all the DNA structures.

Supplementary Table 2. Reaction rate of *Asfv*LIG catalyzed DNA ligation

Enzymes	DNA substrate [a]	K_{obs} ($\times 10^{-6}$, min^{-1}) [b]
WT *Asfv*LIG	DNA-TA	224.82±8.26
WT *Asfv*LIG	DNA-GC	145.42±3.99
WT *Asfv*LIG	DNA-CG	134.83±3.32
WT *Asfv*LIG	DNA-AT	127.94±5.33
WT *Asfv*LIG	DNA-CT	195.11±11.2
WT *Asfv*LIG	DNA-TC	189.94±7.20
WT *Asfv*LIG	DNA-TG	42.84±1.93
WT *Asfv*LIG	DNA-CA	28.69±1.61
WT *Asfv*LIG	DNA-AC	16.44±0.69
WT *Asfv*LIG	DNA-GG	9.67±0.11
WT *Asfv*LIG	DNA-CC	10.38±0.66
WT *Asfv*LIG	DNA-GT	6.83±0.21
WT *Asfv*LIG	DNA-GA	2.83±0.28
WT *Asfv*LIG	DNA-TT	2.49±0.13
WT *Asfv*LIG	DNA-AA	1.96±0.18
WT *Asfv*LIG	DNA-AG	1.44±0.14

[a]: The detailed sequences and structures of DNAs are listed in Table S1.

[b]: Values are means ± s.d. from three independent experiments.

Supplementary Table 3. The DNA ligation rates of the WT and mutant *Asfv*LIG[a].

	WT ($\times 10^{-6}$, min^{-1})	L402R ($\times 10^{-6}$, min^{-1})	Q403F ($\times 10^{-6}$, min^{-1})	Double ($\times 10^{-6}$, min^{-1})	Triple ($\times 10^{-6}$, min^{-1})	Quadruple ($\times 10^{-6}$, min^{-1})
DNA-TA	224.82±8.26	4.41±0.17	2.77±0.13	0.49±0.01	0.65±0.03	0.55±0.03
DNA-GC	145.42±3.99	0.96±0.10	0.92±0.04	0.79±0.03	0.67±0.03	0.52±0.03
DNA-CG	134.83±3.32	1.43±0.15	0.60±0.04	0.65±0.02	0.41±0.02	0.50±0.02
DNA-AT	127.94±5.33	3.05±0.16	2.68±0.07	0.49±0.02	1.45±0.04	1.08±0.04
DNA-CT	195.11±11.20	10.06±0.36	0.31±0.02	0.57±0.04	0.74±0.05	0.22±0.01
DNA-TC	189.94±7.20	8.03±0.26	0.25±0.03	0.61±0.03	0.77±0.04	0.22±0.02

[a]: Values are means ± s.d. from three independent experiments.

Supplementary Table 4. The DNA ligation rates of the WT and mutant *Hs*LIG1[a].

	*Hs*LIG1 ($\times10^{-6}$, min^{-1})	*Hs*LIG1-d ($\times10^{-6}$, min^{-1})	*Hs*LIG1-q ($\times10^{-6}$, min^{-1})
DNA-TA	264.30±12.10	0.35±0.02	n.d.
DNA-AT	236.70±8.43	0.37±0.01	n.d.
DNA-GC	207.30±7.30	0.31±0.02	n.d.
DNA-CG	172.70±5.92	0.71±0.02	n.d.
DNA-CT	37.11±1.32	n.d.	n.d.
DNA-TC	39.81±1.24	n.d.	n.d.
DNA-TG	81.00±3.57	n.d.	n.d.
DNA-GT	29.48±0.90	n.d.	n.d.
DNA-CA	14.91±0.63	n.d.	n.d.
DNA-AC	2.78±0.16	n.d.	n.d.
DNA-GA	0.17±0.01	n.d.	n.d.
DNA-AG	1.60±0.07	n.d.	n.d.
DNA-CT	2.44±0.12	n.d.	n.d.
DNA-TC	12.62±0.31	n.d.	n.d.
DNA-TG	7.76±0.30	n.d.	n.d.
DNA-GT	0.95±0.04	n.d.	n.d.

[a]: Values are means ± s.d. from three independent experiments.

Supplementary Table 5. Sequences of the optimized cDNA of wild type *Asfv*LIG and the primers for *Asfv*LIG mutant constructions

The optimized cDNA sequence of wild type *Asfv*LIG [a] (from 5' to 3')

*GGATCC*ATGCTCAACCAGTTCCCAGGCCAGTATTCTAATAACATTTTCTGCTTCCCGCCAATCGAATCTGAAACGAAAT
CTGGTAAGAAGGCTTCTTGGATTATCTGCGTTCAGGTTGTTCAGCATAACACCATTATTCCAATCACCGACGAGATGTT
CTCTACCGACGTTAAGGACGCGGTTGCGGAAATCTTTACCAAATTCTTCGTTGAGGAAGGCGCGGTGCGTATCTCTAA
AATGACCCGTGTTACCGAAGGCAAGAACCTCGGCAAAAAGAACGCCACTACCGTTGTACACCAGGCGTTCAAAGACG
CCCTGTCTAAGTATAATCGCCATGCGCGTCAGAAACGTGGTGCGCATACCAACCGTGGTATGATCCCGCCGATGCTG
GTTAAATACTTCAATATCATCCCGAAGACGTTTTTCGAAGAAGAAACCGATCCGATTGTGCAGCGTAAACGCAATGGC
GTGCGTGCAGTTGCGTGCCAGCAGGGTGACGGTTGCATCCTCCTGTACTCTCGTACCGAGAAAGAGTTCCTGGGTCT
CGACAACATCAAGAAAGAACTCAAGCAGCTCTATCTGTTCATCGACGTTCGTGTTTATCTGGACGGCGAACTGTACCT
GCACCGTAAACCGCTGCAATGGATCGCGGGTCAGGCGAACGCTAAAACGGATTCTTCTGAACTCCACTTCTACGTTTT
CGACTGCTTCTGGTCTGACCAGCTGCAGATGCCGAGCAACAAACGTCAACAGCTGCTGACCAACATCTTCAAGCAAA
AGGAGGACCTCACGTTCATCCACCAAGTTGAAAACTTCTCTGTTAAGAATGTAGACGAAGCGCTGCGTCTGAAAGCGC
AATTCATTAAAGAAGGTTACGAGGGTGCGATCGTTCGTAACGCGAATGGTCCGTACGAACCGGGTTACAACAACTACC
ACTCTGCGCATCTGGCAAAGCTCAAACCACTGCTCGACGCAGAATTCATCCTCGTGGACTATACCCAGGGTAAAAAAG
GTAAGGACCTGGGTGCAATTCTGTGGGTATGTGAACTGCCGAACAAAAAGCGTTTTGTTGTTACCCCGAAACATCTGA
CCTACGCGGATCGTTACGCGCTGTTTCAAAAACTCACCCCTGCACTCTTTAAGAAGCACCTGTATGGCAAGGAACTGA
CCGTTGAATACGCTGAGCTGTCTCCGAAAACTGGTATCCCTCTGCAGGCGCGTGCGGTTGGCTTCCGTGAACCGATT
AATGTCCTGGAAATCATCTAA*CTCGAG*

Primers used for *Asfv*LIG mutant constructions [b]

Name	Sequence (from 5' to 3')
LIG_F	AAAGGATCCATGCTCAACCAGTTCCCA
LIG_R	AAACTCGAGTTAGATGATTTCCAGGACATTAATCGGTTC
LIG_121F	AAAGGATCCGGTGGTGGTCGTGGTATGATCCCGCCG
LIG_120R	AAACTCGAGTTAGTTGGTATGCGCACCACGTTTCTGACG
LIG_LD_F	TGACCCGTGTTACCGAAGGCGGTGGTACTACCGTTGTACACCAGGC
LIG_LD_R	GCCTGGTGTACAACGGTAGTACCACCGCCTTCGGTAACACGGGTCA
L402R_F	GCTGAACGACGTTGACATGCTGATTATCGTACC
L402R_R	GGTACGATAATCAGCATGTCAACGTCGTTCAGC
Q403F_F	GTACGCGATCTTCTTTTTCACCGGTCCGG
Q403F_R	CCGGACCGGTGAAAAAGAAGATCGCGTAC
N153D_F	CCGTACGCGATCTTCGACTTCACCGGTCCGGTG
N153D_R	CACCGGACCGGTGAAGTCGAAGATCGCGTACGG
L211F_F	CTGCACCGTAAACCGTTCCAATGGATCGCGGGT
L211F_R	ACCCGCGATCCATTGGAACGGTTTACGGTGCAG
LIG_318R	AAACTCGAGTTATTTGAGCTTTGCCAGATGCGCAG

[a]: GGATCC and CTCGAG at the 5'-end and 3'-end are BamHI and XhoI recognition sequence.

[b]: LIG_F and LIG_120R, LIG_121F and LIG_R, LIG_LD_F and LIG_LD_R, LIG_F and LIG_318R, and Q403F_F and Q403F_R were used for the constructions of *Asfv*LIG NTD, *Asfv*LIG ∆N, *Asfv*LIG LD, *Asfv*LIG ∆OB, and Q403F, respectively. Whereas, L402R_F and L402R_R were utilized in the construction of all other mutants.

Supplementary Table 6. Sequences of the primers for WT or mutant *Hs*LIG1 constructions

**Primers used for *Hs*LIG1 mutant constructions [a]**	
Name	Sequence (from 5' to 3')
*Hs*LIG1_F	AAAGGATCCGGTGGTGGTGATCCATCTGGTTACAATCCTGCC
*Hs*LIG1_R	AAACTCGAGTTAGTAGGTATCTTCAGGGTCAGAGCC
*Hs*LIG1_D570N_F	TGCGAATACAAATATAATGGGCAGAGGGCACAG
*Hs*LIG1_D570N_R	CTGTGCCCTCTGCCCATTATATTTGTATTCGCA
*Hs*LIG1_F635L_F	AAGCAGATCCAGCCATTGCAAGTGCTCACCACC
*Hs*LIG1_F635L_R	GGTGGTGAGCACTTGCAATGGCTGGATCTGCTT
*Hs*LIG1_R871L/F872Q_F	AAGGGCATCTCCCTTTTGCAACCTCGGTTTATTCGA
*Hs*LIG1_R871L/F872Q_R	TCGAATAAACCGAGGTTGCAAAAGGGAGATGCCCTT

[a]: *Hs*LIG1_F and *Hs*LIG1_R were used for the construction of WT *Hs*LIG1. *Hs*LIG1_R871L/F872Q_F and *Hs*LIG1_R871L/F872Q_R were used for the constructions of R871L/F872Q double mutant. All other primers were used for the construction of D570N/F635L/R871L/F872Q quadruple mutant.

Supplementary Table 7. Structures and crystallization conditions

<table>
<tr><td colspan="3">DNAs utilized in crystallization</td></tr>
<tr><td>DNA Name</td><td colspan="2">Sequence and secondary structure</td></tr>
<tr><td>DNA1</td><td colspan="2">5′-CCAGTCCGACCCGCATCCCGGA-3′
||||||||||||||||||||||
3′-GGTCAGGCTGTGCGTAGGGCCT-5′
|
P</td></tr>
<tr><td>DNA2</td><td colspan="2">5′-CCAGTCCGACCCGCATCCCGGA-3′
||||||||||||||||||||||
3′-GGTCAGGCTGGGCGTAGGGCCT-5′
|
P</td></tr>
<tr><td colspan="3">Structure and crystallization</td></tr>
<tr><td>Structure Name</td><td>Sample composition</td><td>Well solution</td></tr>
<tr><td>Se-AsfvLIG:DNA</td><td>Se-AsfvLIG (0.15 mM)
DNA1 (0.22 mM)
ATP (1 mM)</td><td>0.1 M BIS-TRIS pH 5.5
15% w/v PEG 10,000
0.1 M Ammonium acetate
0.1 M Cadmium chloride</td></tr>
<tr><td>AsfvLIG:CT1</td><td>AsfvLIG (0.18 mM)
DNA1 (0.2 mM)</td><td>0.1 M BIS-TRIS pH 5.5
15% w/v PEG 10,000
0.1 M Ammonium acetate</td></tr>
<tr><td>AsfvLIG:CT2</td><td>AsfvLIG (0.15 mM)
DNA1 (0.2 mM)
ATP (1 mM)
$MgCl_2$ (2 mM)</td><td>0.1 M BIS-TRIS pH 5.5
15% w/v PEG 10,000
0.1 M Ammonium acetate</td></tr>
<tr><td>AsfvLIG:CG</td><td>AsfvLIG (0.15 mM)
DNA2 (0.18 mM)</td><td>0.1 M BIS-TRIS pH 5.5
10% w/v PEG 10,000
0.1 M Ammonium acetate</td></tr>
</table>

Supplementary Table 8. Data collection and refinement statistics

Structure<br>(PDB ID)	*Asfv*LIG:CT1<br>6IML	*Asfv*LIG:CT2<br>6IMN	*Asfv*LIG:CG<br>6IMK	*Se-Asfv*LIG:DNA<br>6IMJ
**Data collection**[a]				
Space group	$P2_1$	$P2_12_12_1$	$P2_1$	$P2_1$
Cell parameter:				
a (Å)	77.2	58.2	72.3	94.8
b (Å)	53.7	114.0	55.2	63.1
c (Å)	76.2	243.1	79.9	119.3
α (°)	90.0	90.0	90.0	90.0
β (°)	100.5	90	103.1	89.9
γ (°)	90.0	90.0	90.0	90.0
Wavelength (Å)	1.0000	1.0000	1.0000	0.9793
Resolution (Å)	30.0-2.35	30.0-2.70	30.0-2.50	30.0-2.55
Last shell (Å)	2.43-2.35	2.80-2.70	2.59-2.50	2.64-2.55
Completeness (%)	95.5(83.7)	96.7(85.1)	98.5(96.9)	97.7(95.0)
Redundancy	3.9(2.5)	6.8(2.7)	5.5(4.3)	4.7(3.2）
$I/\sigma(I)$	15.9(1.77)	10.9(2)	17.3(2.7)	13.2(1.9)
Rmerge (%)	13.3(50.1)	11.1(39.4)	9.1(48.2)	11.4(46.7)
**Refinement**				
Resolution (Å)	29.9-2.35	29.9-2.70	29.7-2.5	29.9-2.55
R_{work} (%) / R_{free} (%)	21.9/26.8	24.7/26.8	20.5/25.4	22.3/26.1
No. of atoms				
Protein	3140	6438	3268	6288
DNA	895	1787	897	818
water	67	9	100	78
Wilson B factors ($Å^2$)	38.6	40.7	45.1	42.1
Average B factors ($Å^2$)				
Protein	44.6	43.2	49.3	53.5
DNA	43.7	40.4	52.6	79.8
Water	37.9	39.6	44.4	49.7
ATP				76.2
R.m.s. deviations				
Bond length (Å)	0.003	0.004	0.003	0.005
Bond angle (°)	0.490	0.679	0.498	0.765
Ramachandran plot (%)				
Most favored	97.5	95.9	97.5	96.5
Additional allowed	2.5	4.1	2.5	3.5
